# Hydraulic Balance of a *Eucalyptus urophylla* Plantation in Response to Periodic Drought in Low Subtropical China

**DOI:** 10.3389/fpls.2016.01346

**Published:** 2016-09-26

**Authors:** Zhenzhen Zhang, Ping Zhao, Heather R. McCarthy, Lei Ouyang, Junfeng Niu, Liwei Zhu, Guangyan Ni, Yuqing Huang

**Affiliations:** ^1^Key Laboratory of Vegetation Restoration and Management of Degraded Ecosystems, South China Institute of Botany (CAS)Guangzhou, China; ^2^College of Life Sciences, University of Chinese Academy of SciencesBeijing, China; ^3^Guangdong Provincial Key Laboratory of Applied Botany, South China Institute of Botany (CAS)Guangzhou, China; ^4^Department of Microbiology and Plant Biology, University of Oklahoma, NormanOK, USA; ^5^Guangxi Institute of Botany (CAS)Guilin, China

**Keywords:** anisohydric, *Eucalyptus urophylla*, hydraulic balance, stomatal conductance, water use efficiency

## Abstract

A clear understanding of hydraulic regulation in cultivated plants is crucial for addressing challenges to forest water cycling due to climate changes in low subtropical China. Experiments were conducted to determine the hydrologic balance of a *Eucalyptus urophylla* plantation in response to periodic drought. Trees displayed lower stomatal conductance (G_S_) and leaf water potentials (Ψ_L_) during the dry periods. A decrease of 22.4% was found for the maximum reference G_S_ (G_S_ at *D* = 1 kPa; G_Sref-max_). Accordingly, specific hydraulic conductivity (k_s_) decreased by 45.3 – 65.6% from the wet to the dry season, depending on the tree size. Fairly stable leaf stomatal conductance (g_s_) with decreasing Ψ_L_ (Ψ_L_ < -1.6 MPa) contributed to the high water-use efficiency (WUE) of this *Eucalyptus* species. Additionally, the lower stomatal sensitivity (-*m* = 0.53) in the dry season might also be responsible for the high WUE, since we found an anisohydric behavior that was associated with photosynthetically active radiation (Q_0_). Larger trees were found to use water more efficiently than small trees, due to the higher sensitivity of k_s_ to decreasing Ψ_L_. This was also verified by the decreasing leaf carbon isotope discrimination (Δ^13^C) with increasing tree diameter. However, further studies are needed to determine the universality of these results for other Eucalyptus species in this region.

## Introduction

Due to anthropogenic climate changes, the survival of tropical and subtropical forest communities may be threatened in a variety of ways, including increased frequency of severe droughts caused by changes in precipitation pattern (Davidson et al., 2012), especially in the southern region of China (Zhai et al., 2005; Zhou et al., 2011). As previously reported, long term drought may lead to increased tree mortality, and decreased productivity and forest biomass carbon sinks for natural forests (Chaves et al., 2002; Zhou et al., 2013). Recently, planted forests have been suggested to be more vulnerable to severe environmental stress because of their weaker ecological resilience (Bleby et al., 2012).

Under light-saturating conditions and a high vapor pressure deficit (D), most plants reduce stomatal conductance (G_S_) to limit transpiration and to slow down the development of potentially damaging low leaf water potential (Ψ_L_) (Meinzer, 1993), ultimately leading to a decrease in net primary productivity (NPP) (Ryan and Waring, 1992). However, some plants have been hypothesized to follow optimal trajectories to maximize their carbon gain (Hölttä et al., 2011; Rosen, 2013). For example, Eucalyptus species have been reported to have high photosynthetic capacity, WUE, and growth rate (Whitehead and Beadle, 2004), which may imply a high canopy G_S_. These characteristics may minimize and counteract the effects of extreme climate events, and reinforce community resilience (Lloret et al., 2012). However, how these species coordinate decreases in G_S_ to avoid hydraulic failure with the demand to maximize carbon assimilation in dry conditions is poorly understood and less reported.

Generally, plants have been classified into two broad categories based on the ability of stomata to regulate Ψ_L_: isohydric and anisohydric (Jones, 1998; Martínez-Vilalta et al., 2014). Isohydric species adjust their stomatal opening in such a way as to maintain midday Ψ_L_ relatively stable as environmental conditions change. In contrast, anisohydric species have less strict stomatal control, with no discernible threshold of minimum Ψ_L_ (Martínez-Vilalta et al., 2014). It has been found that the slope of the relationship between G_S_ and ln (D) is closely related to the magnitude of G_S_ at D = 1.0 kPa (G_Sref_), and can be used as an empirical relationship to describe isohydric behavior (Oren et al., 1998). The stomatal sensitivity to D of isohydric plants is reported to be linearly proportional to G_Sref_ (i.e., -*m* = 0.6) (Pou et al., 2012), which provides insight into stomatal regulation. Some Eucalyptus (e.g., *Eucalyptus gomphocephala*) have been reported to allow a greater Ψ_L_ range than typical isohydric species and to occupy more drought-prone habitats, since they have xylem that is more resistant to negative water potentials (Franks et al., 2007; West et al., 2008). However, little is known about whether this behavior will facilitate the maximization of carbon assimilation for Eucalyptus.

Except for stomatal regulation, trees mainly respond to soil drought-induced water stress by changes to hydraulic properties, in order to adapt to the environment in the long term (Schäfer et al., 2000; Forrester, 2015). These changes are highly related to stomatal regulation. One of the most commonly observed strategies is that the hydraulic limitation of G_S_ by increasing path length can be mitigated by structural compensation, particularly a reduction in A_L_/A_S_ (Becker et al., 2000; McDowell et al., 2002; Delzon et al., 2004). Some other compensating strategies have been observed, such as larger trees possessing deeper roots and larger conduit diameters (Mokany et al., 2003; Anderegg et al., 2012). Köstner et al. (2002) claimed that the increased path length and gravitational head concomitant with height (H) growth must be offset either by a reduction in G_S_, the ratio of leaf area to sapwood area (A_L_/A_S_), Ψ_L_, or by an increase in hydraulic conductivity (K_s_), to maintain hydraulic balance for any given soil water potential (Ψs) and D. Eucalyptus species usually have large leaf area to support rapid stem growth (White et al., 1998), and thus have high transpiration demands at both the leaf and canopy levels during drought periods (Dawson, 1996). Therefore, we hypothesized that Eucalyptus are likely to maintain a constant G_s_ and support a higher A_L_ as trees grow in order to meet the great growth demands, while other strategies are employed to compensate for the increased hydraulic limitation with tree height and transpiration demand. To shed light on this aspect of Eucalyptus, it is necessary to quantify the effect of tree size on tree growth, as soil water decreases (Feichtinger et al., 2014).

*Eucalyptus urophylla* is the most widely planted forest tree in southern China and its area is still rapidly expanding (Shi et al., 2012). Our study is focused on how fast growing *Eucalyptus* forests in low subtropical China balance hydraulic safety and carbon assimilation under periodical drought. Specifically we asked: (1) How does this species coordinate decreased G_s_ with changes in hydraulic conductivity to maximize carbon assimilation in dry conditions? (2) How do stomatal regulation and hydraulic conductivity converge with tree structural changes so as to maintain fast growth?

## Materials and Methods

### Study Site and Plant Material

This study was conducted at the Huangmian state forest farm (24°66′N, 109°87′E), which is located approximately 60 km southwest of Guilin city in South China. This farm is planted with *E. urophylla* for lumber and pulp production. All of the measurements took place in a 3–5 year old *E. urophylla* stand on a hill with an inclination of approximately 30° facing southwest. The forest density was 1375 trees ha^-1^. The soil of this forest is characterized as heavy loam. This area is characterized by a low subtropical monsoon climate with annual precipitation ranging from 1750 to 2000 mm and an average annual temperature of 19°C. Rainfall is unevenly distributed throughout the year, producing wet (March to September) and dry (October to February of the next year) seasons. Measurements were carried out from June 2012 to May 2013 on 15 trees. The mean tree height and diameter at breast height (DBH) of the sampled trees was 11.5 ± 2.9 m and 10.1 ± 2.2 cm, respectively. An instrument tower 23 m tall was set up within the plantation, providing access to the canopy of the forest stand.

### Sap Flow and Environmental Factors

Self-made Granier-type sensors (20 mm in length; Granier et al., 1996) consisting of a heated (constant heat flow) probe and an unheated thermocouple probe were used to monitor the sap flow density (F_d_, g m^-2^ s^-1^) of the sampled 15 trees. The probes were inserted into the xylem at breast height (1.3 m) on the north side of tree stems. The upper probe was supplied with a constant power of 120 mA. The temperature difference between both probes was measured and converted to F_d_, according to Granier et al. (1996). More details of sensor installation have been described in Zhu et al. (2015) and Zhang et al. (2016). The F_d_ of *E. urophylla* is assumed to be isotropic in terms of evenly distributed leaf transpiration around the tree crown (Burgess and Dawson, 2008). F_d_ was used to estimate transpiration after it was converted into a spatially weighted mean flux based on the radial variation in sap flow density observed in another study of *E. urophylla* (*n* = 38, Zhou et al., 2004). They found that the variation in sapflow density from the outmost of the stem for 3–4 year old *E. urophylla* can be expressed as F_d_ = ax^3^+bx^2^+cx+d, where x is the ratio of the sensor depth to the radial sapwood thickness. We combined the results of the two plots in their study and obtained the equation F_d_ = 4.33x^3^-8.31x^2^+4.07x+0.52. Natural temperature gradients can lead to large potential errors of sap flow measurements (Do and Rocheteau, 2002), however, temperature gradients were found to be negligible in our study (Zhang et al., 2016).

A micro-meteorological station was built on the top of the tower. Photosynthetic photon flux density (Q_0_, μmol m^-2^ s^-1^), temperature (T, °C), relative humidity (RH, %), and wind speed (u, m s^-1^) were measured simultaneously with the sap flow measurements (Zhu et al., 2015; Zhang et al., 2016). Rainfall data (in Guilin) during the study period were obtained from the China Metrological Data Sharing Service System^1^. Soil water content (SWC, m^3^ m^-3^) was monitored with three soil water probes (SM300, UK) that were buried 30 cm under the ground surface.

### Tree Morphological Features

For each sap flow tree, DBH was measured with a diameter tape, and tree height (h) was estimated with a tape dropped from the top of the tower. Leaf area (A_L_) was estimated using an allometric relationship between DBH and the A_L_ that was constructed by harvesting seven trees outside (but near) the experimental plot. During the harvest, five small leaf sub-samples from each tree were scanned with a portable leaf area meter (LICOR-3000, USA) and weighed (fresh weight) to estimate specific leaf area (area/fresh mass ratio). Then all leaves of the harvested trees were collected, weighed and multiplied by the specific leaf area to obtain an estimate of whole tree A_L_. The DBH of the harvested trees ranged from 6.6 to 11.1 cm, while those for sap flow trees ranged from 8.5 to 16.1 cm (i.e., the range of DBH for harvest trees didn’t cover the full range of trees used for sap flow measurements). Thus, we combined our DBH and A_L_ measurements with those from Zhu et al. (2009) (*E. urophylla*; *n* = 9) to derive an A_L_ – DBH relationship [A_L_ = 43.43^∗^ (1-exp (-0.15DBH))^4.93^, *R*^2^ = 0.93, *n* = 16]. Data was obtained from the table in their paper. Sapwood depth and bark thickness were determined from stem cores (5 mm in diameter) obtained with an increment borer from selected trees outside the sap flux measurement plots (5 m away, in the same stand). The sapwood depth was visually distinguished from heartwood based on color, and was used to calculate sapwood area (A_s_), which can be expressed as A_S_ = -0.008 + 0.0015DBH, *R*^2^ = 0.97, *n* = 27. Since sample trees were estimated based on the relationship between DBH and A_s_, we used the fitted relationship between DBH and A_s_ and the A_L_ from the harvested trees together to scale up whole tree transpiration (E_T_). Nocturnal sap flux (E_T-NOC_) was defined as E_T_ that occurred when Q_0_ = 0. Since *Eucalyptus* are reported to have thick, tough and long lived leaves with weak seasonal dynamics (Reich et al., 1999), thus the variation in leaf area would not be accounted for these relationships.

### Hydraulic Properties of Stem Xylem

The physical limitations on water transport in the xylem determine the stomatal behavior and transpiration in trees. This relationship is usually expressed as a combination of Darcy’s law with a simple expression for transpiration which is equated to liquid transport in wood (Whitehead and Jarvis, 1981):

$$G_{Sref}\,\;\alpha \,\;E_{L}\,=\;k_{s}(\Delta \Psi -0.01h)\frac{A_{S}}{A_{L^{h}}}\,\;\;\;\;\;\;\;\;\;\;\;\;\;\;\;\;\;\;\;\;\;\;\;\;\;\;\;\;\;\;\left(1\right)$$

Where k_s_ is the effective hydraulic conductivity from soil to leaves (whole-plant conductance per unit sapwood), and ΔΨ is the water potential difference between root and leaf.

In order to determine the wet-dry seasonal variation in ΔΨ, the leaf water potentials at pre-dawn (Ψ_pre-dawn_, 5:00), pre-night (Ψ_pre-night_, 19:00) and midday (Ψ_midday_, 13:00) were measured with a portable pressure chamber (PMS 1000, Corvallis, OR, USA) on sunny days in the wet (5 days) and dry (4 days) seasons. Five trees were selected for measurement. Ψ_L_ were the mean of three replicate shoots with fully expanded leaves, sampled from the mid-crown of each tree. Since soil moisture variation within a single day is small, Ψ_pre-dawn_ was treated as a substitute for the water potential in the soil (Ψ_s_) (Kim et al., 2008; Bleby et al., 2012). Thus, ΔΨ was estimated as the difference between Ψ_pre-dawn_ and Ψ_midday_.

### Canopy Stomatal Conductance

If forest transpiration is well-coupled with atmospheric factors, the mean stomatal conductance can be estimated based on a simplified equation (Köstner et al., 1992), which is derived from Whitehead and Jarvis (1981). Due to low LAI, the canopy was found to meet the assumptions necessary to adopt this equation (Zhu et al., 2015). It is assumed that the F_d_-scaled transpiration combined with A_s_/A_L_ is a proxy for transpiration rate per unit of leaf area (E_L_). Therefore, mean stomatal conductance (G_s_) for each tree, can be expressed as:

$$G_{S}=(G_{V}T_{a\rho }E_{L})/D$$

where E_L_ is whole-tree transpiration per unit leaf area (g m^-2^ s^-1^), G_V_ is the universal gas constant adjusted for water vapor (0.462 m^3^kPa K^-1^ kg^-1^), T_a_ is the air temperature (K), ρ is the density of water (998 kg m^-3^), and D is in kPa. G_Si_ is in units of mmol m^-2^ s^-1^ (Monteith and Unsworth, 2013).

The forest had an LAI of 1.68 ± 0.28 m^2^ m^-2^ and did not show significant seasonal changes (*p* = 0.78) (Zhu et al., 2015). Therefore, G_Si_ calculation is not subject to errors caused by leaf area dynamics. G_Si_ was estimated after (1) performing a cross-correlation analysis between D and F_d_, and using the most appropriate time lag to implement a time-corrected F_d_ and (2) filtering out data where *D* < 0.6 kPa, in the hours of early morning and late afternoon (Oren et al., 1998).

Along with the Ψ_L_ measurements, we also measured leaf stomatal conductance (g_s_) at mid-day on the abaxial surface of sun-exposed leaves with a steady-state porometer (SC-1, DECAGON, USA). Three leaves of each tree were chosen randomly for these measurements.

### Stomatal Sensitivity to Vapor Pressure Deficit

Stomatal sensitivity is proportionally related to the magnitude of G_s_ at low D (*D* = 1 kPa) when soil moisture is not limiting (Granier et al., 1996), and it can be derived as:

$$G_{S}=G_{Sref}-m\,\ln D$$

where G_Sref_ is the intercept (i.e., the value of G_s_ at *D* = 1 kPa in a log-linear relationship), and -m represents the slope of the regression fit representing stomatal sensitivity to D (i.e., dG_s_/dln D). By analyzing data from a variety of sources, including both porometric and sap flux derived G_s_, -m was demonstrated to be approximately 0.6 (Oren et al., 1998). In our study, the -m and G_Sref_ for *E. urophylla* in the wet and dry seasons was calculated to characterize the response of G_s_ to drought.

A boundary line analysis of the relationship between D and G_s_ was performed for the dry and wet seasons. The datasets of G_s_ for each tree were binned by radiation (nine levels from 0 to 1600 μmol m^-2^ s^-1^). The data at night (*Q*_0_ = 0) were excluded because plant physiological response at night is much more complicated than that in the daytime (Oren et al., 2001). The relationship between the mean lnD and G_s_ of each subset was linearly fitted, and the intercept and slope corresponded to the G_Sref_ (G_s_ at *D* = 1 kPa) and sensitivity in response to D (dG_s_/dlnD, mmol m^-2^ s^-1^ kPa^-1^), respectively (Oren et al., 1998). Then, the relationship between G_Sref_ and -dlnd/dG_s_ for the two seasons was fitted.

In order to determine the radiation regulation of stomatal conductance under different water conditions, the G_Sref_ under different light conditions was normalized by the value of the maximum Q_0_ of each tree in both seasons, and the relationship between the G_Sref_ and mean Q_0_ at that level for all of the trees was fitted with an exponential function expressed as:

$$G_{Sref}=a\times (1-\exp\left(-b\times Q_{o}\right)).$$

Where *a* refers to the maximum dependent variable, i.e., the max G_Sref_ (G_Sref-max_).

### Leaf Stable Carbon Isotopes

After the experiments, we randomly collected leaves at the top of the crown for 11 trees with DBH ranging from 5.2 to 20.4 cm near the sap flow measurement trees. Leaves were dried at 65°C to constant mass, and leaf dry mass was determined to the nearest mg. The dry leaves were then ground to a fine, homogeneous powder (Cernusak and Hutley, 2011). The leaf carbon isotope ratio was determined using a stable isotope ratio mass spectrometer (Isoprime 100, Isoprime, UK) on a subsample of approximately 3 mg leaf material. These analyses were performed in the Public Laboratory of South China Botanical Garden, Guangzhou, China. Carbon isotope discrimination (∆^13^C) in dry leaf matter was calculated as ∆^13^C = (δ^13^Ca - δ^13^Cp)/(1 + δ^13^Cp), where δ^13^Cp is δ^13^C of dry leaf matter, and δ^13^Ca is that of atmospheric CO_2_. We assumed a value of -5.5‰ for δ^13^Ca, according to the previous measurements in low subtropical China (Zou et al., 2009).

### Data Analysis

Boundary-line analysis was conducted in Excel (version 2010, Microsoft Office Excel) to set up the relationship between environmental conditions and maximum canopy stomatal conductance or F_d_. The upper boundary line was derived by: (1) partitioning data of independent variables (V_I_) into specific intervals, (2) calculating the mean and standard deviation of dependent variables (V_d_) in each interval, (3) removing outliers (*P* < 0.05; Dixon’s test), (4) selecting the data falling above the mean plus one standard deviation, and (5) averaging the selected data for each V_I_ interval with *n* ≥ 5 remaining V_d_ values. The intervals with *n* < 5 was excluded to prevent V_I_ intervals with too little information from affecting the relationship.

Statistical analyses were performed using SAS (version 9.2, SAS Institute, Cary, NC, USA). A multiple regression analysis was conducted to determine the effect of hydraulic architecture on tree water use in the wet and dry seasons. A paired *t*-test was used to compare the differences in environmental and plant physiological responses between wet and dry seasons. Origin pro (version 8.6, Origin Lab, USA) was used to draw graphs.

## Results

### Water Conditions and Tree Water Use in Different Seasons

**FIGURE 1:**
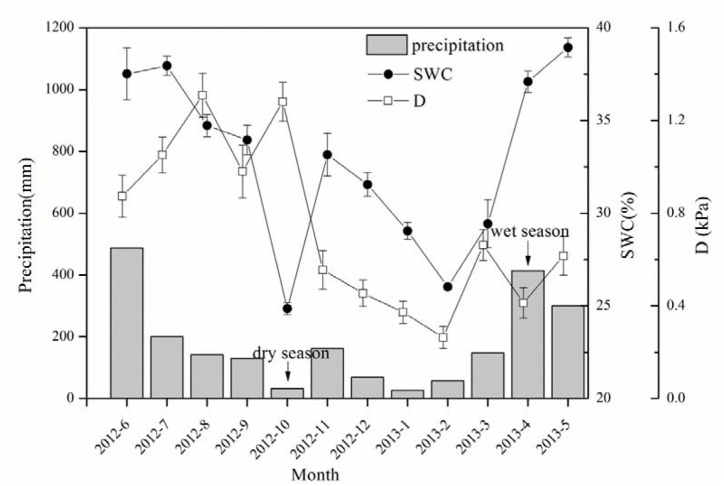
**Precipitation (P), soil water content (SWC) and evaporative demand (D) during the period of sap flow measurement.** SWC data shown in the figure are daily mean ± SE of that month, *n* = 28–31.

The precipitation (P) in the research site totaled 2167.6 mm from June 2012 to May 2013 (**Figure 1**). The water input was mainly contributed by precipitation in late spring and early summer (i.e., from April to June), which accounted for 55.4% of the annual total, while that from October to February (typical dry season in low subtropical China) was only 16%. The SWC in the dry season decreased 32.6% from the wet season, demonstrating a significant difference in the soil water conditions between the wet and dry seasons.

**FIGURE 2:**
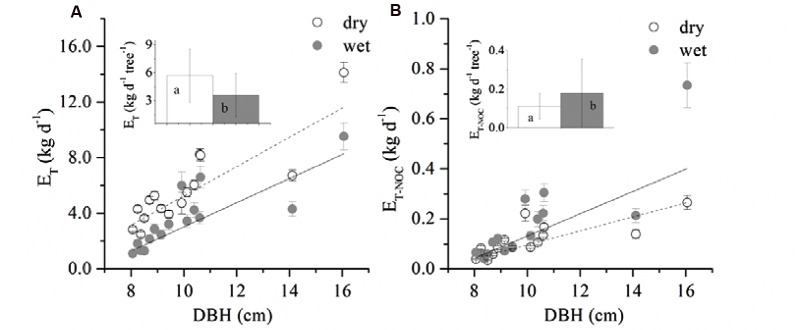
**Relationship between DBH and **(A)** averaged daily transpiration (E_T_), **(B)** averaged total nocturnal sap flow (E_T-NOC_).** Data are mean ± SE, and all linear fittings are significant at the *p* < 0.05 level. The insets in the figure represent the mean E_T_ **(A)** and E_T-NOC_ **(B)** of all 15 trees in dry and wet seasons respectively. Different letters indicate a significant difference between dry and wet seasons.

A boundary line analysis of the relationship between F_d_ and Q_0_ was conducted, and the maximum F_d_ was derived from the exponential relationship. The mean F_d_ of the 15 trees was 41.03 ± 7.97 and 38.82 ± 13.16 g m^-2^ s^-1^ in the dry and wet seasons, respectively, consistent with the pattern of D (**Figure 1**). Overall, F_d_ was not affected by tree size, although it was weakly related to DBH in the dry season (*R*^2^ = 0.19, *p* = 0.06). The wet/dry ratio of F_d_ varied from 0.4 to 0.8 and was not significantly related to the tree size (*R*^2^ = 0.03). Average E_T_ in the dry season (5.7 ± 2.9 kg d^-1^) was 58.0% higher than that in the wet season (3.6 ± 2.3 kg d^-1^) (**Figure 2A**, *p* < 0.01), and linearly increased with tree size (*p* = 0.003). E_T-NOC_ was 0.18 ± 0.021 kg d^-1^ in wet and 0.11 ± 0.01 kg d^-1^ in dry seasons (**Figure 2B**, *p* = 0.047), which contributed 1.82 ± 0.45% and 4.51 ± 1.34% to daily E_T_ in dry and wet seasons, respectively. E_T-NOC_ was also linearly related with tree size (*p* < 0.01).

**FIGURE 3:**
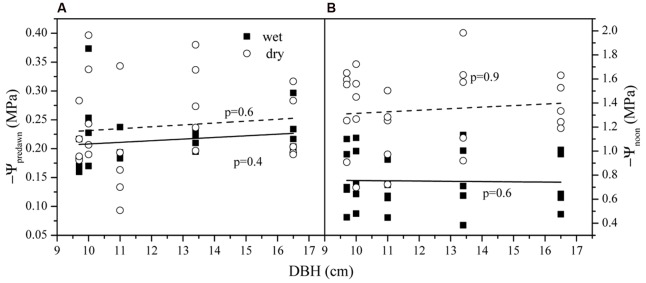
**The leaf water potential at **(A)** pre-dawn (Ψ_pre-dawn_) and **(B)** noon (Ψ_noon_) along the tree size gradient in dry and wet seasons**.

The average Ψ_pre-dawn_ and Ψ_pre-night_ was -0.24 ± 0.04 and -0.29 ± 0.02 MPa in the dry season and -0.21 ± 0.03 and -0.31 ± 0.05 MPa in the wet season. Seasonal differences for both were not significant (*p* > 0.05). The average Ψ_noon_ was higher (-0.75 ± 0.23 MPa) in the wet season than that in the dry season (-1.46 ± 0.23 MPa) (*p* < 0.01). Ψ_pre-dawn_ and Ψ_noon_ versus tree size in both seasons are shown in **Figure 3**. Tree size was not related to variations in either Ψ_pre-dawn_ and Ψ_noon_. Accordingly, the water potential difference at midday (ΔΨ) had a mean of 0.62 ± 0.66 (wet) and 1.22 ± 0.10 MPa (dry). ΔΨ was much higher in wet than in dry season (*p* < 0.01). No difference existed among the five trees for the parameters above (*p* > 0.05).

### VPD Regulated G_s_ under Different Light Conditions

**FIGURE 4:**
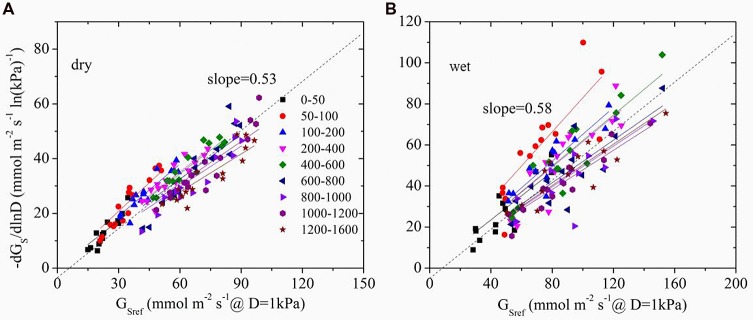
**The sensitivity of average stomatal conductance of tree individuals at each light level in response to increasing vapor pressure deficit (-dG_s_/dlnD) as a function of the canopy stomatal conductance at *D* = 1 kPa (G_Sref_) in dry (A, October) and wet season (B, April).** Different symbols represent the different light levels.

**FIGURE 5:**
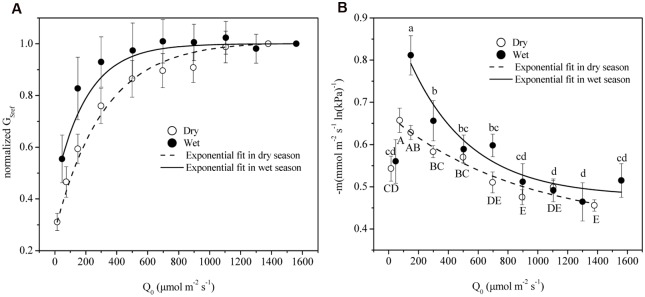
**(A)** G_Sref_ (G_s_ of each tree at D = 1 kPa) normalized based on the highest value in relation to mean photosynthetically active radiation (Q_0_). **(B)** The stomatal sensitivity (–m) of each tree in relation to Q_0_ in dry (open symbols) and wet seasons (solid symbols). Symbols are the mean ± SD of all 15 trees for each light group. Lines are least-square fit through the entire data. Symbols with same letters shown in (a) indicate non-significant differences among light groups, where capital/lower cases refer to dry/wet season.

The time lag between D and G_s_ was found to be 1.3 and 0.5 h in dry and wet seasons respectively. Thus, time-lagged G_s_ was used to calculate G_Sref_. G_Sref_ had a linear relationship with -dG_s_/dLnD in both dry and wet seasons, but significant differences of the slopes were observed under different light levels (ANOVA, *p* < 0.01, **Figure 4**). Normalized G_Sref_ of all of the trees increased rapidly as Q_0_ rose until maximum (**Figure 5A**). G_Sref_ reached 90% of the maximum (G_S90_) when Q_0_ was 287.8 and 167.1 μmol m^-2^ s^-1^ in the dry and wet seasons, respectively. This revealed that G_Sref_ was more sensitive to light in wet season, leading to a lower saturation point than that in the dry season (*p* < 0.01). It was found that -m at different light levels had a weak relationship with tree size (not shown; *p* = 0.33). The effect of Q_0_ on –m was also quantified in both seasons (**Figure 5B**). –m gradually decreased with Q_0_ before a short increase under low light conditions (ANOVA, Duncan, *p* < 0.01), i.e., the sensitivity was not constant within a single day when the light intensity varied substantially. When the data under low light conditions (*Q*_0_< 200 μmol m^-2^ s^-1^) were removed, a linear decrease in -m ranging from 0.32 to 0.83 (dry season) and 0.22 to 1.10 (wet season) with radiation was observed for the 15 trees (**Figure 5B**). Mean -m was substantially higher in the wet season (0.58 ± 0.01) than in the dry season (0.53 ± 0.007) (*p* = 0.038).

Oren et al. (1998) reported that as long as stomata regulate the leaf potential near a constant value, a slope close to 0.6 is expected. The exact slope depends on the *D* range, boundary layer conductance (g_bl_), and changes in hydraulic conductance associated with D. To determine the effect of the *D* range, boundary analysis was conducted with the data shown in **Figure 4** when *D* = 1 kPa – 2 kPa for all light levels. -m derived from this range was compared with that from all of the data, and it turned out to be insignificantly different from the full range in both dry and wet seasons (*p* = 0.43 and 0.14, respectively, ANOVA). In addition, since *E. urophylla* has narrow leaves, g_bl_ in the stand during the wet and dry seasons was found to be 930.1 and 1149.8 mmol m^-2^s^-1^ (unpublished data), respectively, which led to a ratio of g_bl_/G_s_ higher than 2 in the dry season. It is claimed that -m is negatively related to g_bl_/G_s_ and equals 0.55 when the g_bl_/G_s_ is 10 (Oren et al., 1998). Our lower value will produce a higher -m value (>0.55) in the dry season for *E. urophylla*. Thus, it is the changes in hydraulic conductance that are responsible for the -m variation.

### Stomatal Regulation in Relation to Tree Size and Leaf Water Potential

**FIGURE 6:**
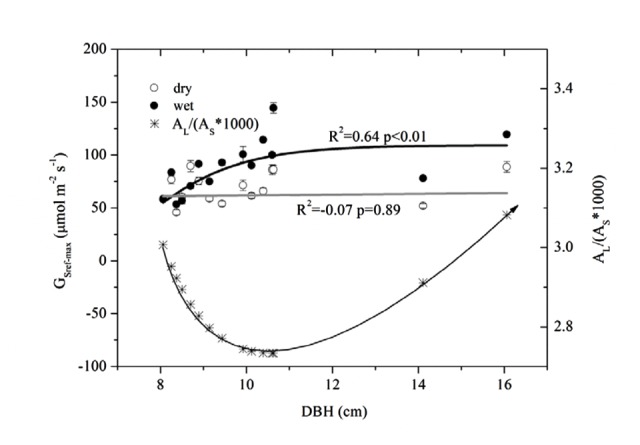
**Relationship between DBH and maximum stomatal conductance at reference D (=1 kPa) (G_Sref-max_) derived from **Figure 4**, and, the A_L_/(A_s_^∗^1000) based on the predicted A_L_ and A_s_ with DBH.** Lines represent least square fits for dry (white circle) and wet (black circle) seasons respectively; data are mean ± SE.

We estimated the max G_s_ (G_Sref-max_) of each tree from the exponential function (equation 4, corresponding to *a*) before it was normalized and fitted to DBH (**Figure 6**). G_Sref-max_ was found to increase with DBH in the wet season before reaching the maximum when DBH > 9 cm. While no clear relationship in the dry season was observed, the mean G_Sref-max_ was higher in the wet season (88.6 mmol m^-2^ s^-1^) than in the dry season (68.8 mmol m^-2^ s^-1^)(*p* < 0.01). The ratio of G_Sref-max_ in the dry to wet season ranged from 0.58 to 1.26 (0.81 on average), decreasing rapidly when DBH < 9 cm and stabilizing when DBH > 9 cm.

**FIGURE 7:**
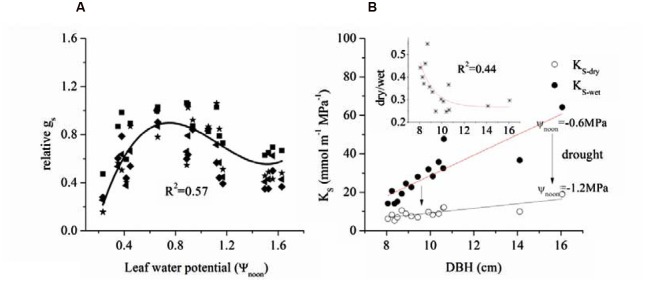
**(A)** Limitation of leaf water potential on relative g_s_ during mid-day (at noon, 12:00-13:00) and **(B)** relationship between tree size and specific hydraulic conductivity k_s_ of 15 sample trees in dry and wet seasons.

We also related Ψ_noon_ with the corresponding mid-day g_s_ (**Figure 7A**) and found a positive relationship between g_s_ and Ψ_L_ when light was low. Based on cell turgor theory, the change in Ψ_L_ is caused by the G_s_-promoted water loss from the leaf (Dow and Bergmann, 2014). However, this value peaked and was maintained from -0.6 to -0.9 MPa before a gradual decrease.

### Hydraulic Conductivity

The combined effects of A_L_/A_s_, H and Ψ_L_ on stomatal conductance of the 15 trees were described using Equation (1) to quantify the tree hydraulic aspects of the plant physiological response (**Table 1**). The A_L_/A_s_ gradually decreased up to approximately DBH = 10 cm, then started to increase when DBH > 11cm (**Figure 6**). We evaluated the seasonal change in k_s_ with Equation (1) when *D* = 1 kPa (**Figure 7B**). It was observed that k_s_ in the wet season was much higher than that in the dry season (29.1 ± 13.1 vs. 9.4 ± 3.2 mmol m^-1^ MPa^-1^). In addition, larger trees had a much higher seasonal variation in k_s_ (*p* < 0.01). As shown in **Figure 7B**, the dry to wet season ratio of k_s_ decreased rapidly with size growth, becoming constant for DBH > 10 cm.

**Table 1:** Parameters that were employed to calculate k_s_ based on Equation 1 for each tree during the dry and wet seasons.

DBH (cm)	h (m)	A_L_/A_s_ (m^2^ m^-2^)	G_s_ (mmol m^-2^s^-1^)		E_L_ (mmol m^-2^ s^-1^)		ΔΨ-0.01 h (MPa)		k_s_ (mmol m^-2^MPa^-1^)	
						
			dry	wet	dry	wet	dry	wet	dry	wet
8.06	12.42	1264.71	58.59	57.98	4.28E-04	4.29E-04	1.08	0.48	6.2	14.2


8.25	12.5	1270.57	76.74	83.72	5.60E-04	6.20E-04	1.08	0.48	8.3	20.7


8.37	13.1	1275.10	45.74	53.41	3.34E-04	3.95E-04	1.07	0.47	5.2	14.1


8.5	13.2	1280.58	60.43	56.84	4.41E-04	4.21E-04	1.07	0.47	7.0	15.2


8.7	13.3	1289.97	89.55	70.67	6.54E-04	5.23E-04	1.07	0.47	10.5	19.2
8.89	13.1	1299.70	75.48	91.55	5.51E-04	6.78E-04	1.07	0.47	8.8	24.6


9.14	14.2	1313.35	58.80	75.08	4.29E-04	5.56E-04	1.06	0.46	7.6	22.6


9.43	14.1	1329.89	53.99	92.89	3.94E-04	6.88E-04	1.06	0.46	7.0	28.1


9.92	14.4	1358.36	71.41	100.73	5.21E-04	7.46E-04	1.06	0.46	9.7	32.0
10.12	14.2	1369.86	61.57	90.18	4.50E-04	6.68E-04	1.06	0.46	8.3	28.4


10.39	14.02	1385.07	65.91	114.34	4.81E-04	8.46E-04	1.06	0.46	8.8	35.7


10.6	14.32	1396.54	86.07	100.21	6.29E-04	7.42E-04	1.06	0.46	11.9	32.5
10.63	14.5	1398.15	86.34	144.45	6.31E-04	0.00107	1.06	0.46	12.1	47.6
14.11	17.7	1514.99	51.96	78.09	3.79E-04	5.78E-04	1.02	0.42	9.9	36.6
16.06	19.4	1518.78	88.80	119.53	6.48E-04	8.85E-04	1.01	0.41	19.0	64.2


## Discussion

### Stomatal Regulation in Response to Leaf Water Potential

The mechanism of stomatal closure is viewed as a direct response to the change in leaf water potential that is related closely to cell turgor (Martorell et al., 2014). In this study, it appears that G_s_ was not held constant in order to maximize carbon assimilation for *E. urophylla* in dry conditions. We found that G_s_ decreased by 22.4% following a two-fold decrease in ΔΨ in the dry season (**Figure 6**). According to equation (2), we assumed that if G_s_ does not change, a 234.4% enhancement of E_L_ or E_T_ would be expected with increased D, since no significant difference in T_a_ and A_L_ was observed between the two seasons (*p* > 0.05). Thus, a 22.4% decrease in G_s_ eventually induced an increase in E_L_ or E_T_ of 159.5%.

That 50∼60% of maximum g_s_ was maintained at -1.6 MPa implies that *E. urophylla* was capable of optimizing carbon assimilation under stressed leaf water conditions. g_s_ peaked between -0.6 and -0.9 MPa after which it decreased gradually (**Figure 6A**), which is similar to the relationship between Ψ_L_ and g_s_ across 70 tree species including Eucalyptus (Klein, 2014). Another example reported by Mielke et al. (2000) showed that G_s_ of *Eucalyptus grandis* maintained ∼40% of the maximum when Ψ_L_ < -2.45 MPa before it reached the minimum Ψ_L_ (-2.8 MPa). It was observed that the stomata maintained 50–60% of the maximum G_s_ when Ψ_L_ reached the minimum (1.6 MPa) (**Figure 7A**), which was consistent with the stabilized G_s_ (∼37.5% of the maximum) when predawn Ψ_L_ < -2.37 for three allopatric *Eucalyptus* species (White et al., 2000). It was shown that the Ψ_L_ has a range of -2.2 MPa to -1.0 MPa when G_s_ decreased to 50% of the maximum for most tree species (Klein, 2014). Thus, it was claimed that the G_s_ of Eucalyptus species tended to be less sensitive to the decrease of ΔΨ than most other woody species. This had already been argued for *Eucalyptus gomphocephala*, since Ψ_L_ is not fixed at or above any particular value (co-varies with monthly rainfall) in a manner that is consistent with typical anisohydric behavior (Franks et al., 2007). Nevertheless, whether this behavior (i.e., not complete stomatal closure under low leaf water potentials) is general among all *Eucalpytus* or not needs further studies (Martorell et al., 2014).

We further quantified this anisohydric behavior via the variation of -m (**Figure 5A**). When light is limited, the increase of Q_0_ will stimulate the opening of stomas as shown in **Figure 4B**, until the threshold is reached. Meanwhile, -m shared the same turning point of Q_0_ with G_Sref_ before gradual decrease. Despite the stabilized G_Sref,_ the stomas became less sensitive to increased D as Q_0_ increased, illustrated by the lower -m, i.e., anisohydric behavior. Thus, light plays a significant role in controlling the stomatal response to D and the carbon assimilation ability. Evidence has shown that taller trees with lower G_Sref_ sensitivity could maintain higher CO_2_ uptake rates over the wide diurnal range of D, which serves to support carbon exchange (Schäfer et al., 2000). Evidently, it is the different perception ability of abscisic acid (ABA) that contributes to the plants isohydric or anisohydric behavior (Tardieu and Simonneau, 1998; Schultz, 2003; Sade et al., 2012), and the release of ABA tends to be activated only when pH is low (such as high Ci) (Ackerson, 1982). For species with higher photosynthetic capacity, lower Ci will be predicted under high light conditions, thus the higher G_s_ and the lower sensitivity to D.

### Hydraulic Conductivity of *E. urophylla*

G_sref-max_ showed a decline of 22.5% from Ψ_L_ = -0.6 MPa to -1.2 MPa, which is less than the 45.3–65.6% decrease of k_s_, implying a more important role of k_s_ regulation in restricting excessive transpiration. As we know, G_s_ as well as k_s_ coincide with decreased Ψ_L_ and start to decrease when xylem water refill cannot not balance canopy water loss (Franks et al., 2007). However, the extent of loss of hydraulic conductivity as Ψ_L_ decreases varies greatly across a variety of species, habitats, and climates (Hacke, 2014). Generally, species growing with high water supply tend to have larger vessels to promote a high hydraulic conductivity in the conducting tissue rather than to minimize the risk of drought-induced xylem embolism (Zach et al., 2010). In *Eucalyptus grandis* and the hybrid of *Eucalyptus grandis × camaldulensis*, vessel diameter and length increased from the dry to wet conditions as water uptake through transpiration increased (February et al., 1995). Because of high annual rainfall in our study site (2167.6 mm, **Figure 1**), growth of the xylem vessel of *E. urophylla* was found to have a mean diameter of 91.4 ± 10.1 μm (Zhao et al., 2014), which favors the significant decrease of k_s_ (45.3–65.6%) in dry conditions.

E_T_ was observed to increase significantly both in dry and wet season with tree size, which implied improved carbon assimilation (Kim et al., 2008). It was found that the changes of A_L_/A_s_ didn’t follow consistent pattern as other studies have reported (increase or decrease, **Figure 6**) (Buckley and Roberts, 2006). However, the G_Sref-max_ in the wet season was observed to increase with tree size (**Figure 6**), and the increased tree height will lead to stomatal regulation (decreased g_s_, Schäfer et al., 2000). Thus, the increased k_s_ with DBH (**Figure 7B**) was thought to contribute to the gradually increased E_T_ and G_s_ (**Figure 2A** and 6). In a tropical old-growth forest, for a variety of species, k_s_ was found to significantly increase with tree height because of the increased mean vessel diameter both in trunk and twig xylem (Zach et al., 2010). Finally, our results also revealed that there was no significant difference of Ψ_L_ among tree sizes (**Figure 3**). Bleby et al. (2012) reported that the decreased Ψ_L_ of *Eucalyptus marginata* usually occurred under natural conditions where resources were limited. At the same time, A_L_/A_s_ was also found to decline in order to maintain hydraulic homeostasis. If the modification of Darcy’s Law for plant water translocation is correct, we can conclude that the increased burden on water transport due to increased G_s_, A_L_/A_s_ and tree height were well compensated by the increased k_s_. However, increased k_s_ will lead to more vulnerable xylem in the face of drought stress (Thomas et al., 2004; Ladjal et al., 2005). As observed in **Figure 7B**, k_s_ decreased 45.3 and 65.6% respectively for the smallest and largest tree, which implies a lower resistance for suppressed trees with decreasing Ψ_L_ (Ambrose et al., 2009).

### Enhanced WUE with Moderate Periodical Drought

**FIGURE 8:**
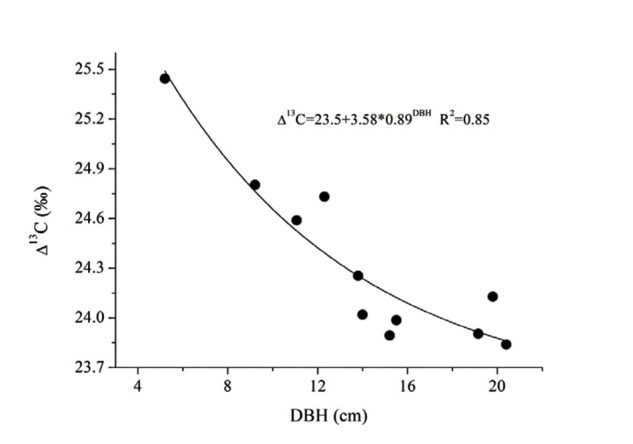
**Relationship between DBH and the leaf carbon isotope discrimination (^13^C) of 11 trees for *E. urophylla***.

The substantial increase of transpirational demand in the dry season led to the decrease of k_s_ and G_s_ (**Figure 7B**). We found that G_s_ decreased by 22.4% from the wet to dry seasons, while k_s_ decreased by 45.3–65.6% more than G_s_. If leaves in both seasons have the same demand for CO_2_, the WUE should be higher in the dry season. Such a water-use strategy may contribute to high water-use efficiency for *E. urophylla*, especially under dry conditions because water flux in the xylem is reduced more than G_s_ in the leaves. This possibility had been observed in some other studies (Brienen et al., 2011; Maseyk et al., 2011; Liu et al., 2012). In addition, the k_s_ ratio of dry/wet decreased from 0.55 to 0.30 with increased tree size (**Figure 7B**), implying a higher decrease in water loss for large trees. This meant that *E. urophylla* in our stands tended to improve their WUE in the dry period. To verify this hypothesis, we conducted leaf carbon isotope analysis along the tree size gradient. Consistently, Δ^13^C showed a clear decrease with DBH (**Figure 8**). A number of studies have argued that moderate drought favors high WUE of tree species from different ecosystems, including forest and desert (Maseyk et al., 2011; Liu et al., 2012). Otto et al. (2014) argued that dominant clonal *Eucalyptus grandis* × *urophylla* trees use water more efficiently compared with native species. However, the underlying mechanism responsible for higher WUE is unclear. Our results indicated possible mechanisms for high WUE in dominant trees, which might be verified by further studies on the annual net biomass yield/annual transpiration, since isotope discrimination only provides an estimate of intrinsic WUE rather than the ratio of uptaken CO_2_ to actual fluxes of water vapor.

## Conclusion

In a moist low subtropical area in South China with periodic drought, *E. urophylla* was observed to close stomata under lower water supply conditions with decreased Ψ_L_, but showed anisohydric behavior with gradually stabilizing high G_s_ at low Ψ_L_, especially under high light conditions (deceased -m). k_s_ decreased 45.3–65.6% from wet to dry season, which is significantly higher than the proportional decline of G_s_ (22.4%) and may be responsible for the high WUE of Eucalyptus species. As tree size increased, greater sensitivity of k_s_ to water loss was synchronous with improved WUE (decreased Δ^13^C), which contributed to the constant stomatal conductance in the dry season (G_Sref-max_) and the persistent increase of A_L_/A_s_ with DBH and insignificant change in Ψ_L_ among trees for maintaining the hydraulic balance. We are uncertain of the generality of the above behavior for other Eucalyptus species, which deserves further studies.

## Author’s Note

We declare that the previous version of this manuscript “Water use strategies of a young *Eucalyptus urophylla* forest in response to seasonal change of climatic factors in South China” submitted to Biogeosciences Discussion (doi: 10.5194/bgd-12-10469-2015) and appearing as a preprint service, was finally rejected.

## Author Contributions

ZZ and PZ organized and supported the entire study. HM, LO, JN, LZ, GN, and YH performed the sap flow and physiological measurements. ZZ also wrote this manuscript, PZ and HM edited this manuscript.

## Conflict of Interest Statement

The authors declare that the research was conducted in the absence of any commercial or financial relationships that could be construed as a potential conflict of interest.

## Footnotes

1 http://data.cma.cn/
